# Neuropeptides Substance P and Calcitonin Gene Related Peptide Accelerate the Development and Fibrogenesis of Endometriosis

**DOI:** 10.1038/s41598-019-39170-w

**Published:** 2019-02-25

**Authors:** Dingmin Yan, Xishi Liu, Sun-Wei Guo

**Affiliations:** 1 Shanghai OB/GYN Hospital, Fudan University, Shanghai, 200011 China; 2 Shanghai Key Laboratory of Female Reproductive Endocrine-Related Diseases, Fudan University, Shanghai, China

## Abstract

Endometriotic lesions are known to be hyperinnervated, especially in lesions of deep endometriosis (DE), which are frequently in close proximity to various nerve plexuses. DE lesions typically have higher fibromuscular content than that of ovarian endometriomas (OE) lesions, but the underlying reason remains elusive. Aside from their traditional role of pain transduction, however, whether or not sensory nerves play any role in the development of endometriosis is unclear. Here, we show that, thorough their respective receptors neurokinin receptor 1 (NK1R), calcitonin receptor like receptor (CRLR), and receptor activity modifying protein 1 (RAMP-1), neuropeptides substance P (SP) and calcitonin gene related peptide (CGRP) induce epithelial-mesenchymal transition (EMT), fibroblast-to-myofibroblast transdifferentiation (FMT) and further turn stromal cells into smooth muscle cells (SMCs) in endometriotic lesions, resulting ultimately in fibrosis. We show that SP and CGRP, or the rat dorsal root ganglia (DRG) supernatant, through the induction of NK1R and CGRP/CRLR/RAMP-1 signaling pathways, promoted EMT, FMT and SMM in endometriosis, resulting in increased migratory and invasive propensity, cell contractility, production of collagen, and eventually to fibrosis. Neutralization of NK1R and/or CGRP/CRLR/RAMP-1 abrogated these processes. Extended exposure of endometriotic stromal cells to SP and/or CGRP or the DRG supernatant induced increased expression of α-SMA, desmin, oxytocin receptor, and smooth muscle myosin heavy-chain. Finally, we show that DE lesions had significantly higher nerve fiber density, increased staining levels of α-SMA, NK1R, CRLR, and RAMP-1, concomitant with higher lesional fibrotic content than that of OE lesions. The extent of lesional fibrosis correlated positively with the staining levels of NK1R, CRLR, and RAMP-1, as well as the nerve fiber density in lesions. Thus, this study provides another piece of evidence that sensory nerves play an important role in promoting the development and fibrogenesis of endometriosis. It explains as why DE frequently have higher fibromuscular content than that of OE, highlights the importance of lesional microenvironment in shaping the lesional fate, gives more credence to the idea that ectopic endometrium is fundamentally wounds that go through repeated tissue injury and repair, and should shed much needed light into the pathophysiology of endometriosis.

## Introduction

Characterized by the ectopic deposition and growth of endometrial-like tissues, endometriosis is an estrogen-dependent and inflammatory disorder affecting ~8% of premenopausal women^1^. However, this seemingly innocuous definition camouflages the disease that can manifest a wild variation in size, location, color, depth of infiltration, presence or absence of adhesion, and the proportion of endometriotic epithelial/stromal cells, let alone a kaleidoscopic variation in symptomology and severity. It has been widely accepted that there are three major subtypes of endometriosis: ovarian endometriomas (OE), deep endometriosis (DE), and superficial peritoneal endometriosis (PE)^2^. Based mainly on their different histology, these subtypes have long been hypothesized to be three *separate* disease entities and perhaps have different pathogenesis and pathophysiology^2^.

Previously called deep infiltrating endometriosis^3,4^ but now redefined as adenomyosis *externa* or simply deep endometriosis^5,6^, DE is less prevalent than OE^7^ and is found not only in the rectovaginal septum, but also in all fibromuscular pelvic structures such as the uterosacral and utero-ovarian ligaments and the muscular wall of pelvic organs^6^. DE includes rectovaginal lesions as well as infiltrative forms that involve vital structures such as bowel, ureters, and bladder^8^. Though less prevalent than OE, >95% of women with DE complain of severe pain, including dysmenorrhea, dyspareunia, non-menstrual pelvic pain, and, less commonly, dyschezia and dysuria^5,8^, and DE is the most difficult subtype of endometriosis to manage clinically^9–12^.

Research on DE has been remarkably extensive, yet its pathogenesis and pathophysiology still remain elusive^5,6,8,13,14^. One feature that DE stands out from other subtypes of endometriosis is its presence of smooth muscle metaplasia (SMM) and its high degree of fibrotic tissues^4,15–17^, which explains the choice of the term adenomyosis externa, presumably because of its enriched fibromuscular content^15^ akin to adenomyosis. This is one of several reasons that prompted a recent proposal to re-define endometriosis to include the pro-fibrotic nature of endometriosis^18^.

Despite all the vast phenotypic variation in different subtypes of endometriosis, however, all subtypes as well as adenomyosis have one defining hallmark in common, namely, they all go through cyclic or repeated bleeding similar to eutopic endometrium^19^. Consequently, they are essentially wounds that go through repeated tissue injury and repair (ReTIAR)^20,21^. As a result of this ReTIAR, the ectopic endometrium actively interacts with various cells in its microenvironment, activates the transforming growth factor (TGF)-β1/Smad3 signaling and experiences epithelial-mesenchymal transition (EMT) and fibroblast-to-myofibroblast transdifferentiation (FMT), causing increased collagen production and cellular contractility and eventually resulting in fibrosis^22^. Extended exposure to TGF-β1 also results in elevated expression of α-smooth muscle actin (α-SMA) and of markers of terminally differentiated smooth muscle cells (SMCs) in the stromal component of endometriotic lesions, accounting for SMM that is common in endometriotic lesions^23–26^. This essentially depicts the natural history of endometriotic lesions^27^.

In support for this notion, we recently found that, compared with OE, DE lesions appeared to have gone through EMT, FMT, and SMM more thoroughly and more extensively, and, accordingly, exhibited significantly more fibromuscular tissues yet reduced cellularity and vascularity^28^. The issue left unresolved is why there are such differences between OE and DE.

In contrast to OE lesions, DE lesions are in uncanny proximity to several nerve plexuses such as inferior hypogastric, vesical, uterovaginal, and rectal plexus. More strikingly, DE lesions are frequently hyperinnervated^29–34^. This raises the prospect that sensory nerve-derived neuropeptides such as substance P (SP) may precipitate the development and fibrogenesis of endometriosis. Indeed, we found, via mouse models, that chemical denervation of sensory nerves resulted in significantly less lesional fibrosis than denervation of sympathetic nerves^35^. In addition, surgical denervation of sensory nerves significantly reduced lesional fibrosis, and the antagonism of neurokinin 1 receptor (NK1R), the SP receptor, also achieved the same effect^35^.

Consequently, we hypothesized that SP and calcitonin gene related peptide (CGRP), another neuropeptide secreted by sensory nerves, induce EMT and FMT in endometriosis, leading to increased collagen production and eventually to fibrosis. In addition, extended exposure to SP and CGRP as well as sensory dorsal root ganglia (DRG) supernatant further turn endometriotic stromal cells into differentiated smooth muscle cells, yielding SMM. This study was undertaken to test these hypotheses.

## Results

### SP and CGRP induce morphological changes consistent with EMT in endometriotic epithelial cells

**Figure 1:**
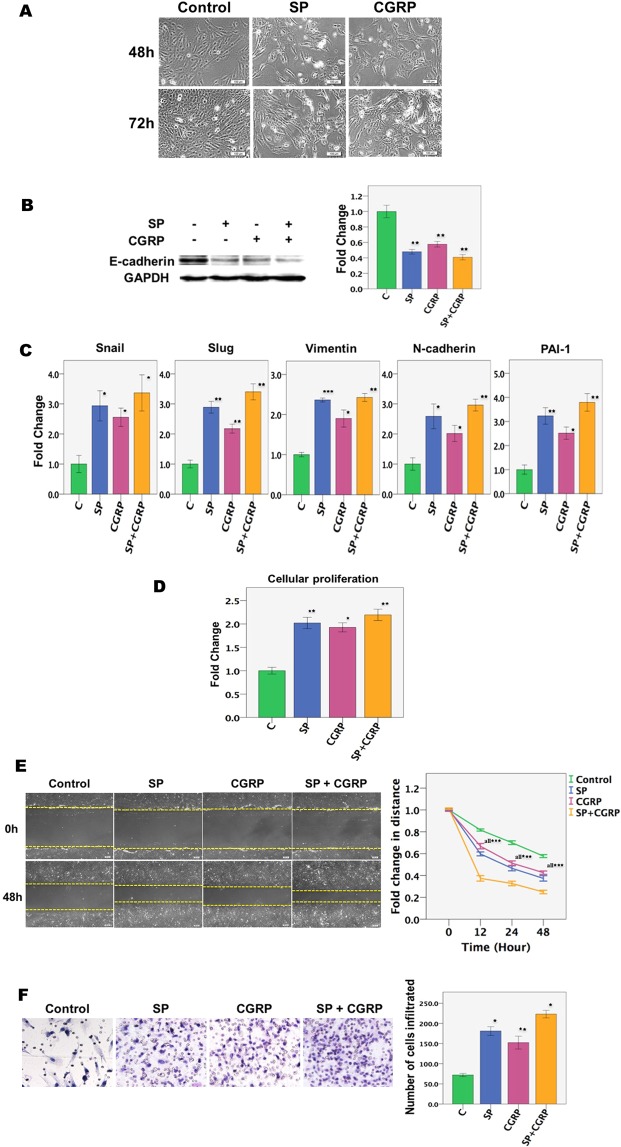
The effect of SP and CGRP treatment on morphological, molecular and functional changes in endometriotic epithelial cells. **(A)** Representative micrographs of endometriotic epithelial cells (11Z) treated with SP or CGRP for the indicated time. In all experiments, the concentration of both SP and CGRP was 10^−7^ M unless indicated otherwise. Scale bar = 100 μm. (**B**) Left panel: Detection of protein levels of E-cadherin by immunoblotting of lysates of 11Z cells treated vehicle, SP, CGRP, or both SP and CGRP for 72 hours (n = 3). The grouping of blots from the same protein were not cropped, and all protein blots were from the same gel. Right panel: Bar plot summarizing the fold changes in E-cadherin protein expression levels after treatment with SP and/or CGRP. **(C)** Relative fold change in gene expression of Snai1, Slug, vimentin, N-cadherin and PAI-1 in 11Z cells treated with indicated conditions for 72 hours (n = 3). Values are normalized to GAPDH expression. Right panel: Relative fold change of the protein levels of E-cadherin in 11Z cells (n = 3). (**D**) Proliferation of 11Z cells treated with SP or CGRP for 72 hours was evaluated by the CCK-8 assay (n = 6). (**E**) The migratory capacity, as evaluated by the scratch assay, of 11Z cells treated with SP or CGRP was significantly increased as compared with untreated ones. The cells were photographed at 0, 12 and 24 h after being scratched (n = 6). The distance between edges of cells traversed was calculated, in pixel numbers, relative to the initial scratch distance. Scale bar = 100 μm. (**F**) The representative photomicrographs of the invaded 11Z cells in transwell assay under different treatments for 72 h. The total number of cells invaded to the bottom of the transwell was then counted (n = 5). Magnification: × 200. Scale bar = 100 μm. Symbols of statistical significance: *p < 0.05, **p < 0.01, ***p < 0.001. Data are represented in mean ± SD. C: Control; SP: Substance P; CGRP: calcitonin gene related peptide.

We first evaluated the effect of SP and/or CGRP treatment on 11Z cells, an endometriotic epithelial cell line^36^. We found that 11Z cells treated with SP or CGRP for 72 hours underwent conspicuous morphological changes consistent with EMT, changing the morphology from round-shaped to spindle-like feature and dispersed (Fig. 1A). Consistent with the morphological changes, the protein expression levels of E-cadherin, an epithelial marker, were significantly decreased after 72 hours of SP or CGRP treatment (Fig. 1B), while the expression levels of genes involved in EMT, such as Snai1 and Slug, and of markers of mesenchymal cells, such as vimentin and N-cadherin, as well as the gene involved in fibrosis such as PAI-1 (Serpine1) were significantly elevated in 11Z cells treated with SP and/or CGRP as compared with the controls (all p-values < 0.05; Fig. 1C).

### SP and CGRP enhance the proliferative, migratory and invasive propensity in endometriotic epithelial cells

By CCK-8 cell proliferation and viability assay, we found that 11Z cells treated with SP and/or CGRP for 72 hours resulted in significantly increased cellular proliferation as compared with controls (all p-values < 0.05; Fig. 1D). Using the scratch assay, we found that the migratory capability of 11Z cells treated with SP and/or CGRP was significantly increased as compared with untreated ones (all p-values < 0.001; Fig. 1E). Moreover, we found, by invasion assay, that treatment with SP and/or CGRP for 72 hours significantly increased the invasiveness of endometriotic epithelial cells as compared with controls (all p-values < 0.05; Fig. 1F).

### SP and CGRP induce FMT in endometriotic epithelial cells

**Figure 2:**
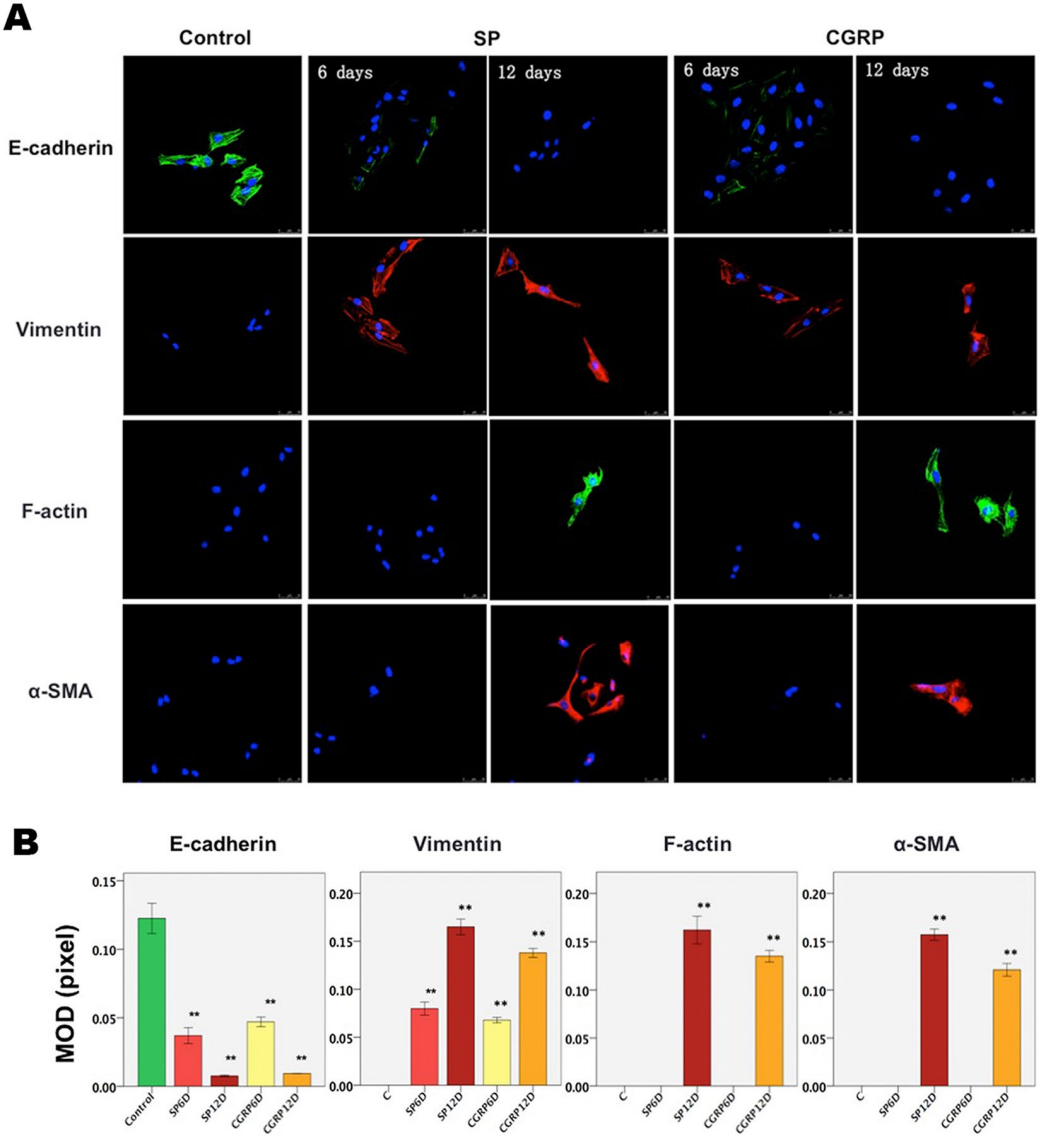
SP and CGRP induce transdifferentiation to a myofibroblast phenotype in endometriotic epithelial cells. Immunofluorescence evaluation of the expression of E-cadherin, vimentin, α-SMA and F-actin in 11Z cells after treatment with SP or CGRP for indicted times. (**A**) Representative photomicrographs of 11Z cells showing immunofluorescent staining of E-cadherin (in green), vimentin (in red), α-SMA (in red) and F-actin fibers (in green) after treatments with SP (10^−7^ M) or CGRP (10^−7^ M) for 6 and 12 days, respectively. The nucleus was stained blue. The control group was also evaluated at day 0 before the treatment, and no change was found treated with vehicle for the same durations. Since the results were identical to the cells evaluated at 0 days, the figures are not shown. Magnification: ×400. Scale bar = 50 μm. (**B**) Summary of the immunofluorescence results by mean optical density (MOD) (in pixels) on the same exposure condition. **p < 0.01. Data are represented in mean ± SD.

Consistent with the EMT-like changes in endometriotic epithelial cells treated with SP or CGRP, we also found, through laser scanning confocal microscopy, that the expression of E-cadherin in 11Z cells was substantially reduced when treated with SP or CGRP as compared with controls, especially when treated for an extended period (Fig. 2). In addition, vimentin expression became progressively elevated (all p-values < 0.01; Fig. 2), indicating that both SP and CGRP induced EMT in endometriotic epithelial cells.

Not surprisingly, endometriotic epithelial cells initially did not show any expression of α-SMA or F-actin (Fig. 2). However, their expression became conspicuous when treated with SP or CGRP for 12 days (all p-values < 0.01; Fig. 2), suggesting that, after a prolonged exposure to SP or CGRP, the endometriotic epithelial cells were further transdifferentiated from mesenchymal cells through EMT to myofibroblasts. In contrast, cells treated with vehicle for the same durations were all negative for vimentin, α-SMA and F-actin but positive for E-cadherin as shown in Fig. 2A (only the results at day 0 were shown since the remaining results were identical to the cells evaluated at day 0).Thus, we provided another piece of evidence that SP or CGRP secreted by sensory nerves promote EMT-like morphological and molecular changes, and extended exposure of endometriotic epithelial cells to SP or CGRP also promote myofibroblast activation.

### SP and CGRP induce FMT and further differentiate endometrial and primary endometriotic stromal cells into smooth muscle cells (SMCs)

**Figure 3:**
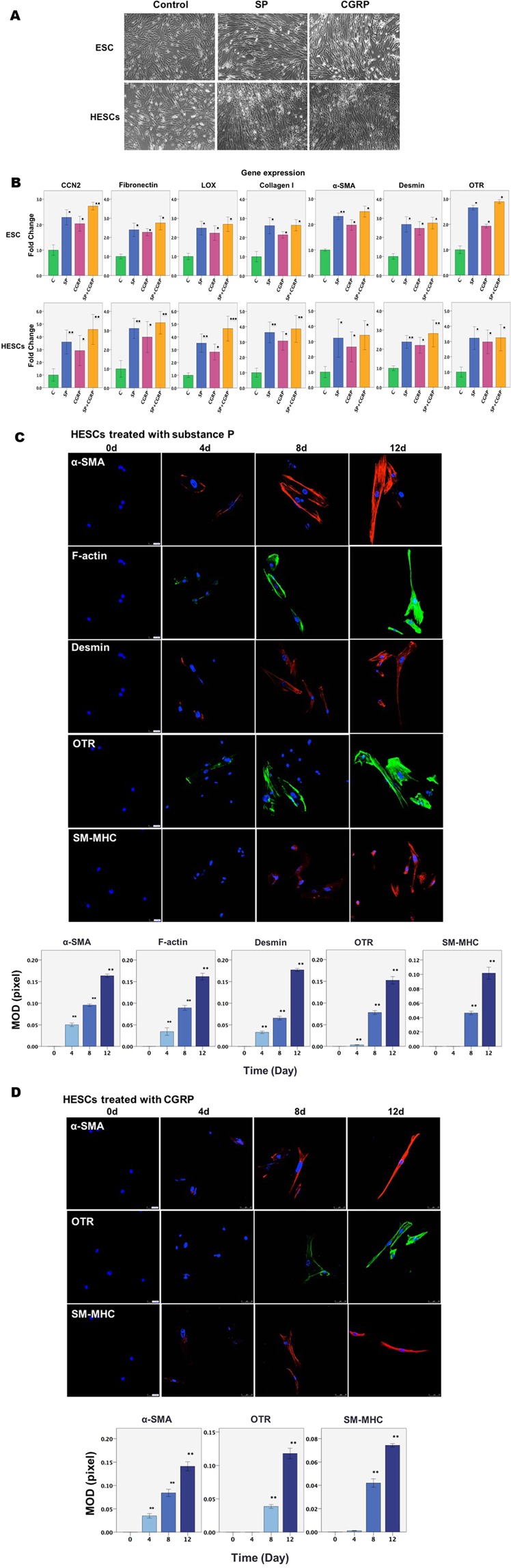
The effect of SP and CGRP on morphological and molecular changes in an endometrial stromal cell line (ESCs) and primary endometriotic stromal cells (HESCs). (**A**) Representative morphology of ESCs and HESCs treated with SP or CGRP for 72 h. Scale bar = 100 μm. (**B**) Relative fold change of gene expression levels of CCN2 (CTGF), FN, LOX, Collagen I (COL1A1), α-SMA, desmin and OTR in ESCs and HESCs treated with vehicle, SP, CGRP, or both SP and CGRP for 12 days (triplicates for ESCs and n = 8 for HESCs). Values are normalized to the GAPDH expression. (**C**) Upper panel: Immunofluorescence results showing progressively increased staining of α-SMA, F-actin, desmin, OTR and SM-MHC in HESCs treated with SP as the duration of treatment increases. In contrast, HESCs treated with vehicle for the same durations showed no change in the staining levels of either α-SMA, F-actin, desmin, OTR or SM-MHC and, as such, the results were identical to the treated cells evaluated at day 0 and were thus not shown. Lower panel: Summary of the immunofluorescence results for HESCs treated with SP for different durations by MOD (in pixels). (**D**) Upper panel: Immunofluorescence results showing progressively increased staining of α-SMA, OTR and SM-MHC in HESCs treated with CGRP as the treatment duration increases. The untreated cells at the same time points showed no change at all time points and were identical to the treated cells evaluated at day 0, and, as such, the results are not shown. Lower panel: Summary of the immunofluorescence results for HESCs treated with CGRP for different durations by MOD (in pixels). Scale bar = 50 μm. Symbols of statistical significance: *p < 0.05; **p < 0.01, ***p < 0.001. Data are represented in mean ± SD. In all experiments, the concentration of both SP and CGRP was 10^−7^ M.

We next investigated the effect of SP and/or CGRP on endometrial and endometriotic stromal cells in the development of endometriosis. We found that ESCs and HESCs treated with SP and/or CGRP underwent noticeable morphological changes reminiscent of myofibroblast transdifferentiation, since the morphology of these cells went from spindle like and became thinner and more elongated reminiscent of muscle fibers and further dispersed (Fig. 3A).

α-SMA is considered as a marker for myofibroblast and SMC^37^. Oxytocin receptor (OTR) is detected only in fully differentiated SMCs^38^, desmin is a marker for differentiated and mature SMC^37^, and smooth muscle-myosin heavy chain (SM-MHC) is considered as a marker restricted for the SMC^39^. Since myofibroblasts are the most important effector cells in fibrogenesis^40^, we next evaluated the expression of genes known to be involved in FMT in ESCs and HESCs. Treatment with SP and/or CGRP resulted in significant upregulation of CCN2 (CTGF), FN, LOX, COL1A1, and α-SMA in both ESCs and HESCs as compared with that in controls (all p-values < 0.05; Fig. 3B). More remarkably, when ESCs and HESCs were exposed to SP and/or CGRP for an extended time (12 days), the protein expression levels of α-SMA and of desmin and OTR, the two markers for differentiated SMCs, were significantly increased as compared with that treated with vehicle (Fig. 3B).

In addition, immunofluorescence confirmed the progressively increased expression of α-SMA, F-actin, desmin, OTR and SM-MHC in HESCs treated with SP as the treatment duration increased (Fig. 3C), suggesting prolonged exposure of HESCs to SP and/or CGRP results in SMCs-like differentiation (Fig. 3C,D). In contrast, HESCs treated with vehicle for the same durations showed no change in the staining levels of either α-SMA, F-actin, desmin, OTR or SM-MHC and, as such, the results were identical to the treated cells evaluated at day 0 and were thus not shown. These results strongly indicate that SP and/or CGRP secreted from sensory nerves induce FMT and further SMM in endometriotic stromal cells.

### SP and CGRP increase cellular contractility and collagen production in endometriotic and normal endometrial stromal cells after FMT

One important step in wound healing is the wound contraction, which is accomplished by myofibroblasts^41^. Collagen gel contraction by fibroblasts has been ascribed to the contraction of actin filaments, which generate the cumulative traction force exerted by fibroblasts on collagen fibrils^42^. *In vitro* collagen gel contraction is considered to simulate the wound contraction process *in vivo*^43^.

**Figure 4:**
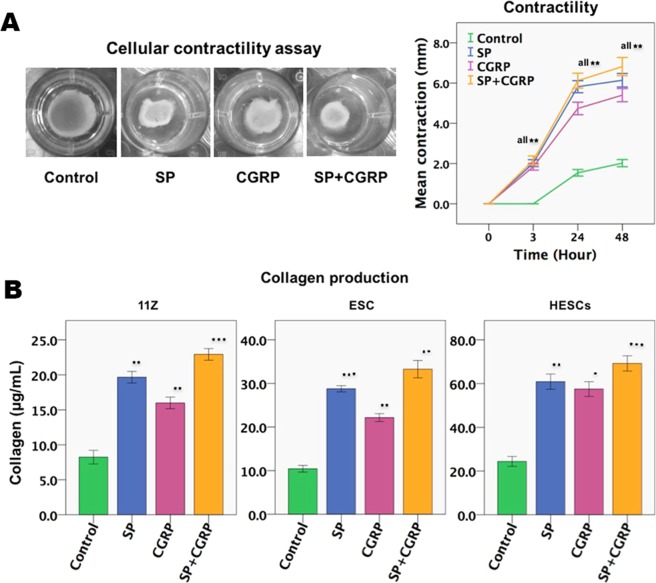
SP and CGRP increase cellular contractility and promote collagen production in endometriotic cells. (**A**) Left panel: the representative results of collagen gel contraction assay for primary endometriotic stromal cells (HESCs) after treatments with SP (10^−7^ M) or CGRP (10^−7^ M) for 48 h. Right panel: Summary of the contractility experiment for HESCs, in terms of diameter of the gel surface, measured at 0, 3, 24, and 48 h, respectively (n = 5). (**B**) The amount of soluble collagen secreted by 11Z cells (n = 3), ESCs (n = 3) and HESCs (n = 8) after treatment with SP and/or CGRP for 72 h. The absorbance value was determined at 570 nm (optical density, or OD), and the concentration of collagen (μg/mL) was determined by the collagen reference standard curves. Data are represented in mean ± SD. Symbols of statistical significance: *p < 0.05; **p < 0.01, ***p < 0.001.

We found that, compared to that treated with vehicle, the contractility of 11Z cells, HESCs, and ESCs was all significantly increased after treatments with SP and/or CGRP (all p-values < 0.05) just for 3 hours and further increased progressively and then plateaued at 48 hours (similar results for 3 cell types, but only results on HESCs are shown in Fig. 4A). In addition, and consistent with FMT and increased contractility, treatment with SP and/or CGRP for 72 hours resulted in significantly increased production of soluble collagens in 11Z cells, ESC and HESCs as compared with controls (all p-vales < 0.05; Fig. 4B). These results clearly show that treatment with SP and/or CGRP led to myofibroblast activation and increased collagen production in endometriotic epithelial and stromal cells as well as normal endometrial stromal cells.

### The DRG supernatant induces fibroblast transdifferentiation to myofibroblasts and further to SMC

**Figure 5:**
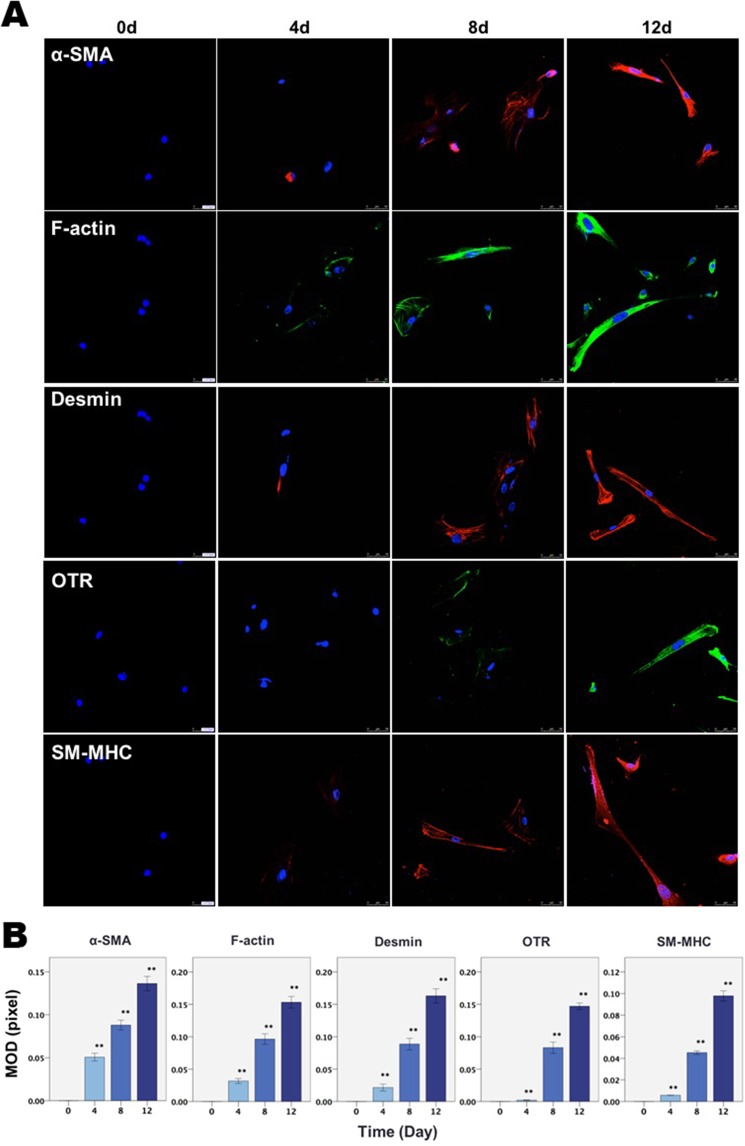
Immunofluorescence results showing the expression of myofibroblast and smooth muscle cell markers in HESCs treated with DRG supernatant for different durations as indicated. (**A**) Representative photomicrographs showing that DRG supernatant gradually induces the transdifferentiation of primary endometriotic stromal cells to a myofibroblast and then smooth muscle cell like phenotype. After treatment with the DRG supernatant for 12 days, the staining levels of α-SMA, F-actin, desmin, OTR and SM-MHC were significantly elevated. In contrast, HESCs treated with just medium for the same durations showed no change in the staining levels of either α-SMA, F-actin, desmin, OTR or SM-MHC and, as such, the results were identical to the treated cells evaluated at day 0 and were thus not shown. Scale bar = 50 μm. (**B**) Summary of the immunofluorescence results for HESCs treated with DRG supernatant for different durations by MOD (in pixels). Data are represented in mean ± SD. **p < 0.01.

Considering that SP and/or CGRP, which are secreted mostly from sensory nerves, induced FMT and SMM in endometriotic stromal cells as shown above, we next investigated whether co-culture with the DRG supernatant can also induce these cells to undergo differentiation into SMCs as shown above. By immunofluorescence, we found that HESCs treated with the DRG supernatant for 4 days had slightly increased α-SMA and F-actin staining but, not surprisingly, negative staining of OTR, desmin, and SM-MHC. However, OTR, desmin and SM-MHC staining was progressively and significantly elevated as the treatment duration increased, concomitant with progressively increased α-SMA and F-actin staining (Fig. 5). In contrast, HESCs treated with just medium for the same durations showed no change in the staining levels of either α-SMA, F-actin, desmin, OTR or SM-MHC and, as such, the results were identical to the treated cells evaluated at day 0 and were thus not shown. Consistently, the gene expression levels of α-SMA, OTR, desmin and SM-MHC were progressively and significantly elevated as the duration of treatment increased, indicating that sensory nerves induce endometriotic stromal cells to undergo further differentiation into fully differentiated SMCs and are thus responsible for SMM in endometriotic lesions.

### SP and CGRP neutralization reverses DRG-induced EMT and FMT in endometriotic cells

**Figure 6:**
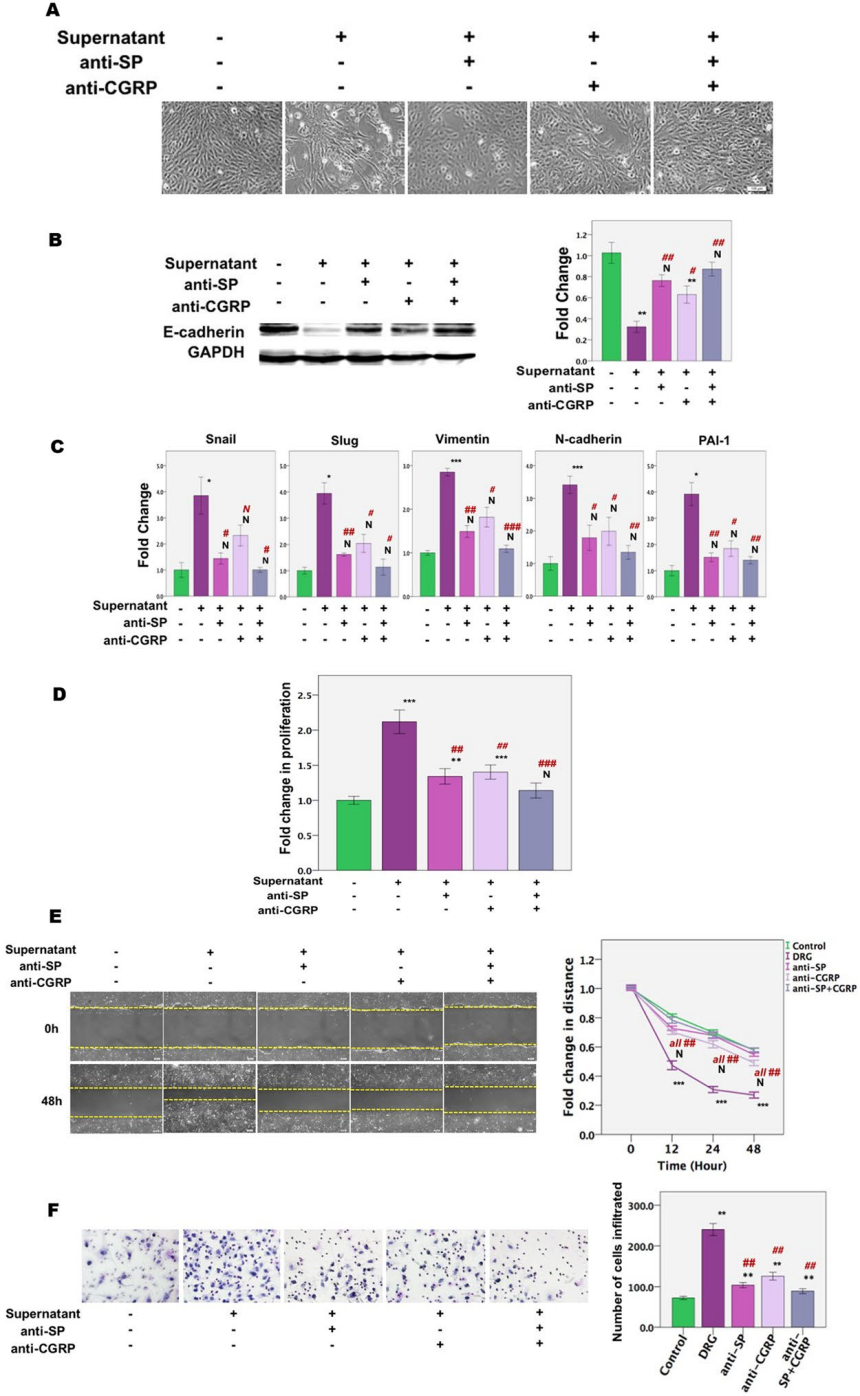
SP and CGRP neutralization reverses DRG-induced EMT and FMT in 11Z cells. (**A**) Representative morphology of 11Z cells treated with medium, the DRG supernatant with pre-treatment with aprepitant (10^−6^ M), CGRP fragment 8–37 (10^−6^ M), or both aprepitant (10^−6^ M) and CGRP 8–37 (10^−6^ M), or without for 12 days. Scale bar = 100 μm. (**B**) Left panel: Detection of protein levels of E-cadherin by immunoblotting of lysates of 11Z cells treated with indicated condition for 12 days (n = 3). The grouping of blots from the same protein were not cropped, and all protein blots were from the same gel. Right panel: Relative fold change in protein levels of E-cadherin in 11Z cells treated with indicated condition for 12 days (n = 3). (**C**) Relative fold change of gene expression levels of Snai1, Slug, vimentin, N-cadherin, and PAI-1 in 11Z cells treated with indicated conditions for 12 days (n = 3). Values are normalized to the GAPDH expression levels. (**D**) Results of SP and CGRP neutralization on cellular proliferation of 11Z cells, as measured by CCK-8 assay (n = 8). The 11Z cells were treated with the indicated conditions for 12 days. (**E**) Results of SP and CGRP neutralization on cell migratory capacity, as evaluated by the scratch assay, of 11Z cells treated with indicated conditions for 12 days. The cells were photographed at 0, 12, 24 and 48 hours, respectively, after being scratched. The distance between two edges that cells traversed was calculated relative to the initial scratch distance as measured with pixel values. Scale bar = 100 μm. (**F**) Results of SP and CGRP neutralization on invasiveness. The representative photomicrographs of the invaded 11Z cells in the transwell assay after indicated treatments (Magnification: × 200). Cells, after 12 days’ indicated treatments, were added to the top of transwells coated with Matrigel and treated as indicated. The total number of cells invaded to the bottom of the transwell was then counted. Scale bar = 100 μm. * or ^#^p < 0.05, ** or ^##^p < 0.01, *** or ^###^p < 0.001; N: not statistically significant (p > 0.05). Data are represented in means ± SDs. Symbols of statistical significance: *, **, and *** indicate different significant levels when compared with the untreated cells, while ^#, ##^, and ^###^ indicate different significant levels when compared with the cells treated with the DRG supernatant. * or ^#^p < 0.05; ** or ^##^p < 0.01; *** or ^###^p < 0.001.

Given the roles of SP and CGRP in inducing EMT, FMT and SMM in endometriotic cells as shown above, we next investigated whether SP and CGRP neutralization, respectively, by Aprepitant, a potent and selective NK1R antagonist, and CGRP Fragment 8–37, a selective competitive antagonist of CGRP receptors, would abolish sensory nerve-induced EMT, FMT, and SMM in endometriotic cells. We found that neutralization of SP and/or CGRP significantly abrogated DRG-induced changes in morphology and the expression of genes/proteins involved in EMT (with the only exception of Snail when CGRP was neutralized, likely due to the lack of statistical power) in endometriotic epithelial cells, especially when SP and CGRP were both neutralized (Fig. 6A–C). In addition, neutralization of either SP, CGRP or both significantly and nearly completely abolished sensory nerve-induced proliferative, migratory and invasive propensity in endometriotic epithelial cells (Fig. 6D–F).

**Figure 7:**
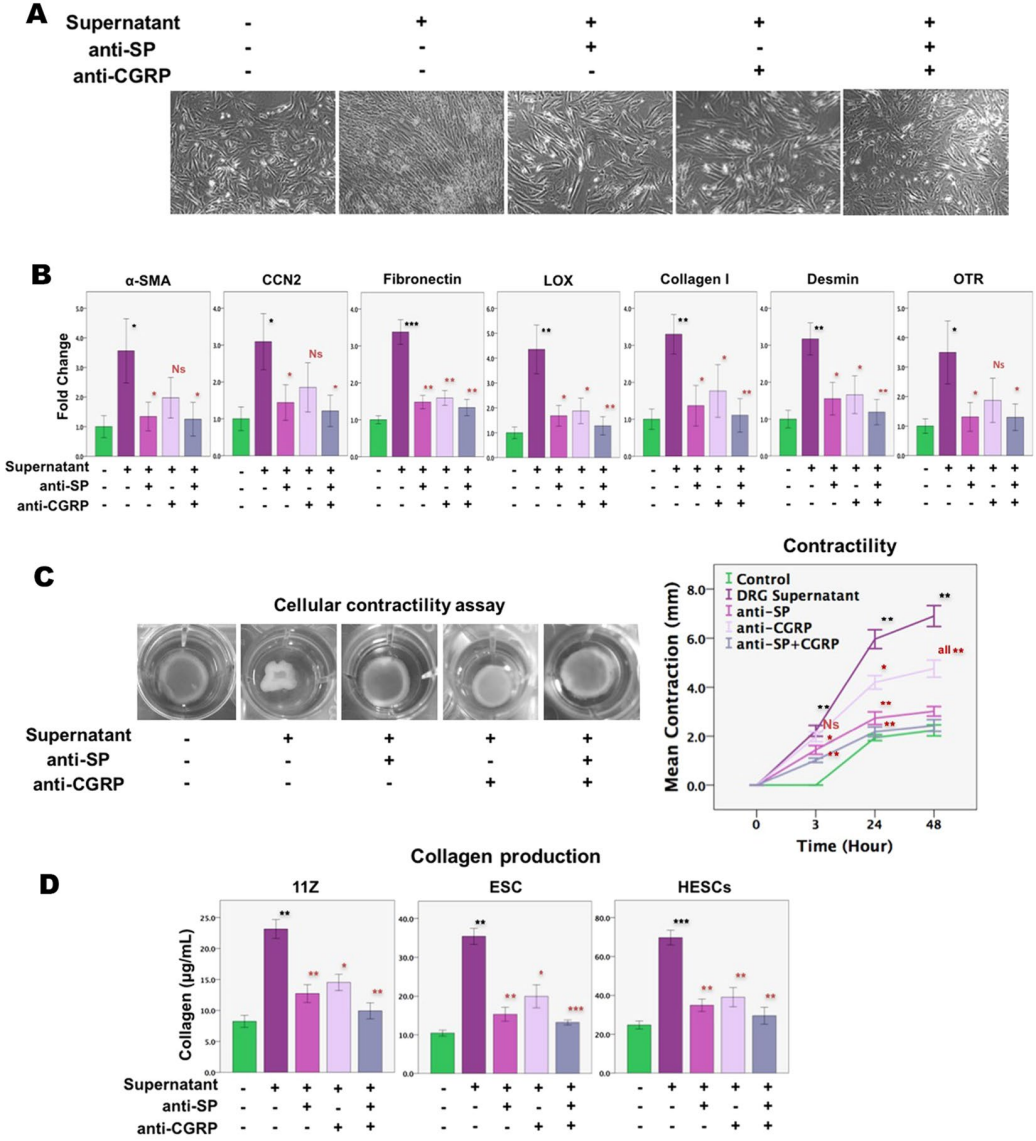
SP and/or CGRP neutralization completely abolished sensory nerve-induced FMT and further SMM in primary endometriotic stromal cells. (**A**) Representative morphology of HESCs treated with medium, DRG supernatant with pre-treatment with aprepitant (10^−6^ M), CGRP 8–37 (10^−6^ M), or both or without for 12 days. Scale bar = 100 μm. (**B**) Relative fold change of gene expression of α-SMA, CCN2 (CTGF), FN, LOX, COL1A1, desmin and OTR in HESCs treated with indicated conditions for 12 days (n = 8). (**C**) Left panel: SP and/or CGRP neutralization abolished increased cellular contractility induced by DRG supernatant treatment for 12 days in endometriotic stromal cells (HESCs). Right panel: Summary of the contractility experiment for HESCs, in terms of diameter of the gel surface, measured at 0, 3, 24, and 48 h, respectively, after release (n = 5). (**D**) The amount of soluble collagen produced by11Z cells (n = 3), ESCs (n = 3) and HESCs (n = 8) after treatment with indicated conditions for 12 days. The absorbance value was determined at 570 nm (optical density, or OD) and the concentration of collagen (μg/mL) was determined by the collagen reference standard curves. Data are represented in mean ± SD. Symbols of statistical significance: * or ^#^p < 0.05, ** or ^##^p < 0.01, *** or ^###^p < 0.001; N: not statistically significant (p > 0.05) Note that *^,^ **, and *** are the statistical significance levels when compared with the untreated cells, while ^#, ##^, and ^###^ indicate statistical significant levels when compared with the cells treated with the DRG supernatant.

Similarly, with the only exception of α-SMA, CCN2, and OTR, for which CGRP neutralization did not yield statistically significant reduction likely due to lack of sufficient statistical power, SP and/or CGRP neutralization completely abolished DRG-induced FMT and SMM as manifested by changes in morphology (Fig. 7A) and the expression levels of markers of myofibroblasts and of SMCs in endometriotic stromal cells (Fig. 7B). Consistent with the immunofluorescence results shown above (Fig. 5), the treatment of the DRG supernatant significantly increased the gene expression levels of α-SMA, desmin and OTR (Fig. 7B). Moreover, consistent with the changes induced by SP and/or CGRP treatment, neutralization of SP and/or CGRP also abolished increased cellular contractility induced by the DRG supernatant in endometriotic stromal cells, and abolished the production of soluble collagens induced by the treatment with the DRG supernatant in endometriotic epithelial and stromal cells as well as endometrial stromal cells (Fig. 7C–D).

### The extent of lesional fibrosis correlates with nerve fiber density and lesional NK1R/RAMP-1/CRLR expression levels

Having demonstrated the effect of sensory nerve-derived SP and/or CGRP in promoting progression of endometriotic lesions through EMT, FMT, and SMM via *in vitro* and *in vivo* studies, we now turn to human endometriotic lesions. We predicted that lesional expression levels of NK1R, the SP receptor, as well as calcitonin receptor like receptor (CRLR) and receptor activity modifying protein 1 (RAMP-1), the two CGRP receptors, correlate with the extent of lesional fibrosis.

We first performed an IHC analysis of α-SMA, NK1R, RAMP-1 and CRLR in both OE and DE tissue samples. We also evaluated the density of CGRP + sensory never fibers within the lesions as well as the extent of lesional fibrosis by Masson trichrome staining. Since the expression of RAMP-1 and CRLR, the two receptors for CGRP, has not been reported in endometrium or endometriosis, we also stained the two for normal endometrial tissues.

**Figure 8:**
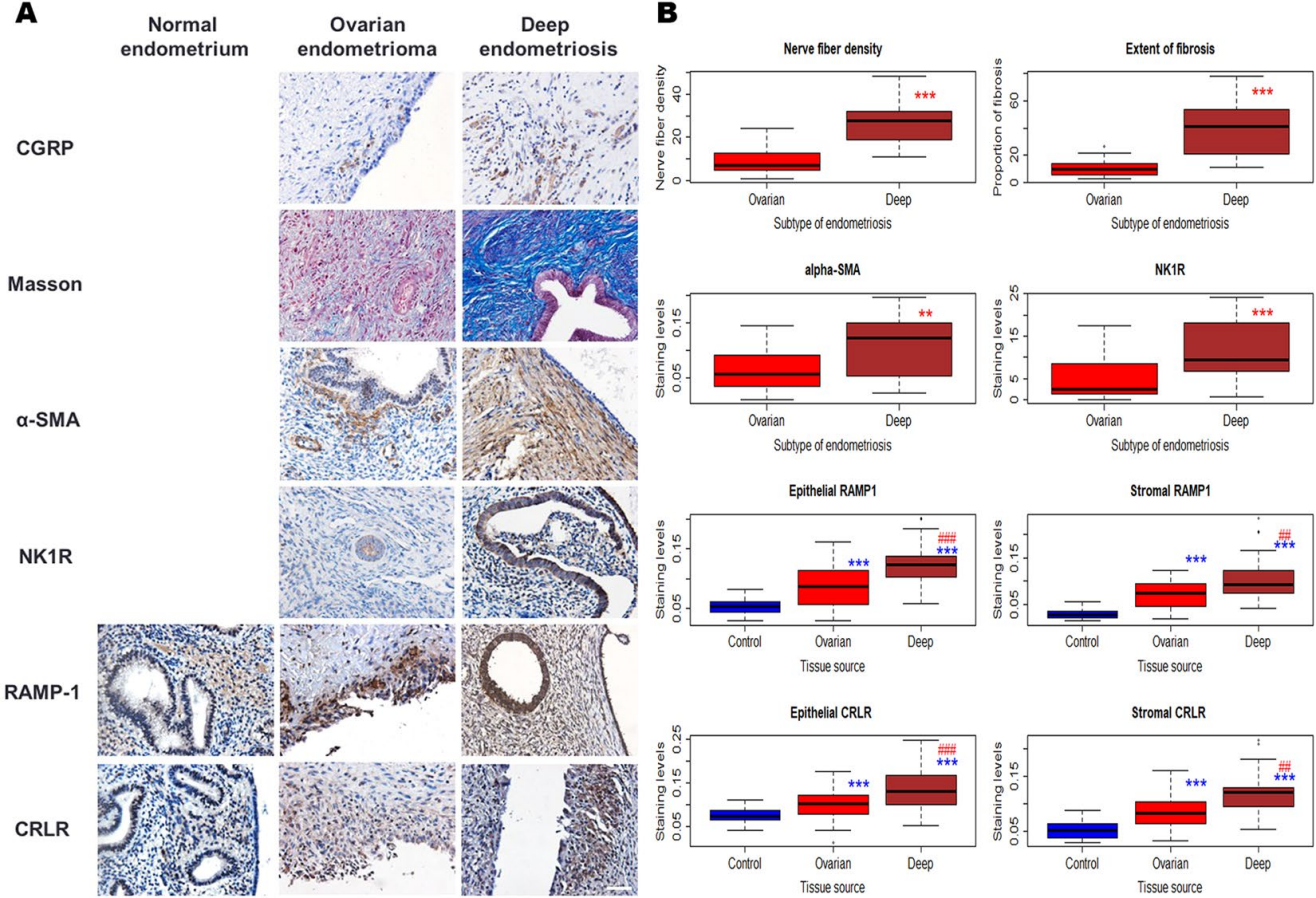
Immunohistochemistry evidence of fibrosis and nerve fiber density in endometriotic lesions. (**A**) Representative photomicrographs of immunohistochemistry analysis of α-smooth muscle actin (α-SMA), neurokinin 1 receptor (NK1R), calcitonin gene related peptide (CGRP) and substance P (SP), along with Masson trichrome staining in ovarian endometriotic lesions and in DIE lesions. Magnification: ×400. Scale bar = 50 μm. (**B**) Immunostaining levels of CGRP-positive nerve fibers, Masson staining, α-SMA and NK1R. Scatter plot shows the relationship between the nerve fibers density via CGRP immunoreactivity staining and extent of fibrosis via Masson trichrome staining in both ovarian endometriotic and DIE lesions, with its color corresponding to the pain stage of patients. **p < 0.01, ***p < 0.001. Data are represented in means ± SDs.

We found that the immunoreactivity against α-SMA was seen mostly in cytoplasm in endometriotic stromal cells, as expected, and NK1R staining was seen mostly in cytoplasm and membranes in endometriotic epithelial cells (Fig. 8A). The lesional density of CGRP positive nerve fibers correlated with the pain severity in women with endometriosis (Spearman’s r = 0.85, p < 2.2 × 10^−16^). Concomitantly, lesional staining levels of α-SMA, NK1R, RAMP-1 (either epithelial or stromal), and CRLR (either epithelial or stromal) correlated with the sensory nerve fiber density in the lesions (all r’s > 0.69, p < 8.7 × 10^−10^). As shown with Masson trichrome staining, the extent of fibrosis was also remarkably aggravated in endometriotic lesions as nerve fiber density increased, especially in DE lesions (Fig. 8B). The RAMP-1 and CRLR immunostaining could be found in both epithelial and stromal components of normal endometrium, but their staining levels were significantly lower than that in either OE or DE lesions (Fig. 8A,B).

As expected and also consistent with previously reported^28,44^, we found that, compared with OE lesions, DE lesions have significantly higher nerve fiber density, higher extent of fibrosis, and higher α-SMA and NK1R staining levels (Fig. 8). In addition, the staining levels of both RAMP-1 and CRLR in both OE and DE lesions were significantly higher than that of normal endometrium irrespective of cellular component (Fig. 8). Linear regression analysis indicated that, after controlling for age, menstrual phase, parity, presence or absence of adenomyosis, and presence or absence of uterine fibroids, the RAMP-1 and CRLR staining levels were still significantly higher in OE and DE lesions than that of normal endometrium (all p-values < 0.036, all *R*^2^ ≥ 0.34). For endometriotic lesions, the NK1R, RAMP-1, and CRLR staining levels were significantly higher in DE lesions than OE lesions, even after the adjustment for age, menstrual phase, parity, presence or absence of adenomyosis, and presence or absence of uterine fibroids (all p-values < 0.008).

**Figure 9:**
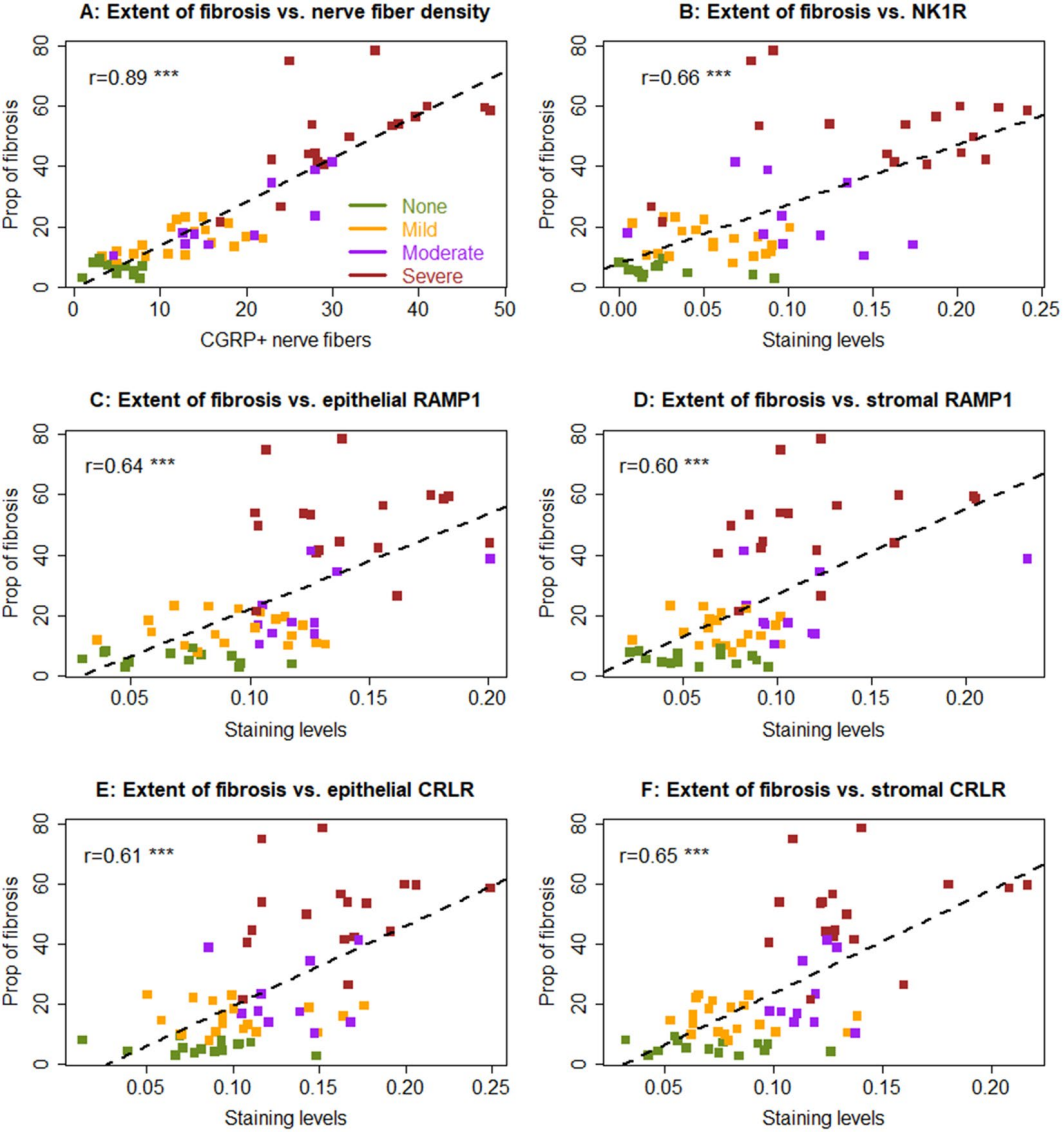
Scatter plot of the extent of lesional fibrosis and lesional nerve fiber density (**A**), lesional staining levels of NK1R (**B**), epithelial RAMP-1 (**C**), stromal RAMP-1 (**D**), epithelial CRLR (**E**) and stromal CRLR (**F**). Each dot in the figure represents one data point (one patient) and different colors represent different severity of dysmenorrhea. The numbers shown in each figure is the Pearson’s correlation coefficient, and *** indicates that the statistical significance level of the correlation coefficient is less than 0.001.

Not surprisingly, the severity of dysmenorrhea correlated positively with the nerve fiber density in lesions (Spearman’s r = 0.85, p < 2.2 × 10^−16^; Supplementary Fig. 1A). It also positively correlated with the extent of fibrosis in endometriotic lesions (r = 0.90, p < 2.2 × 10^−16^; Supplementary Fig. 1B), and the lesional α-SMA and NK1R immunostaining levels (r = 0.81, p = 2.3 × 10^−15^, and r = 0.71, p = 1.8 × 10^−10^; Supplementary Figure 1C,D), as well as with that of RAMP-1 and CRLR (Supplementary Fig. 1E–H). As expected, the extent of lesional fibrosis positively correlated with the lesional staining levels of NK1R, RAMP-1, and CRLR (all r’s ≥ 0.60, p < p < 5.2 × 10^−17^; Fig. 9).

## Discussion

In this study, we have shown that, as with platelet-derived TGF-β1^22^, sensory nerve-derived neuropeptides SP and CGRP facilitate EMT, FMT and differentiation to SMC in endometriosis, yielding increased collagen production, elevated cellular contractility, and eventually fibrosis. Neutralization of their respective receptors, such as NK1R, RAMP-1 and CRLR, however, abrogates these processes. Extended exposure to sensory DRG supernatant further turned endometriotic stromal cells into differentiated SMCs, resulting in SMM. More remarkably, lesional nerve fiber density correlated with the lesional expression levels of NK1R, RAMP-1 and CRLR, and ultimately with the extent of lesional fibrosis as well as the severity of pain in women with endometriosis. These data, taken together with our *in vivo* data^35^ in conjunction with the successful establishment of a mouse DE model of by infusion of SP and CGRP in addition to i.p. endometrium injection^45^ provide an additional strong piece of evidence that sensory nerve fibers play a potent facilitatory role in expediting the development and fibrogenesis of endometriotic lesions. This also provides an answer to a long-standing conundrum as why DE lesions have abundant SMC-like cells^17,25,46^ and more extensive fibrosis than that of OE lesions^2,4,24,28,46^.

Considering the consensus that DE patients suffer much more serious pain, these results may explain the differences between OE and DE that sensory nerve fibers and their secreted neuropeptides accelerate the lesional development through facilitating EMT, FMT and SMM and ultimately fibrosis, making the DE lesions that are typically more fibromuscular than that of OE lesions.

Viewed from the lens of the ReTIAR^22^, our results are consistent with the well documented roles of SP/CGRP and their receptors in wound healing and fibrogenesis. Successful repair of injured tissues requires diverse interactions among different cells, biochemical mediators, and the cellular microenvironment^47–49^. It is well documented that sensory denervation impairs cutaneous wound healing through increased apoptosis and reduced proliferation^50,51^. Indeed, SP acts as an immune modulator and injury messenger in various peripheral tissues^52^. Furthermore, SP mobilizes mesenchymal stem cells^52^ and endothelial progenitor cells (EPCs)^53^ in the bone marrow, and induces them to migrate into the injured peripheral tissues where they are involved in tissue regeneration. SP accelerates the normal acute and chronic wound healing processes^54–56^. Subcutaneous administration of SP accelerates the normal acute wound healing response via increased angiogenesis, resulting from SP-mediated EPC mobilization^57^. Similarly, genetic deletion or blockade of CGRP receptors has been suggested to be detrimental to wound healing^58–60^. SP can indirectly promote hemangiogenesis in tissues by recruiting granulocytes with angiogenic potential from the blood circulation^61^. As both SP and CGRP are vasodilators^62^, the activation of the SP/CGRP and their receptors would result in plasma extravasation^63,64^ and platelet extravasation, contributing to platelet aggregation in endometriosis as we demonstrated previously^65^.

NK1R is expressed in human and rodent uterus^66–69^, so are CRLR and RAMP1^70^. The expression of NK1R and SP-coding gene TAC1 is reported to be upregulated by estrogen^68,71^ and TNF-α^44^. This may suggest that increased local production of estrogen and proinflammatory cytokines may induce NK1R in myometrium, causing uterine hyperactivity and, subsequently, pain, as in colon^72,73^. As local production of TNF-α and estrogen is increased in endometriotic lesions, TAC1 and NK1R expression would be and in fact has been reported to be elevated^44^. NK1R activation has been shown to be involved in ERK1/2 protein (MAPK), p38 MAPK, NF-κB, PI3K, Akt, Src, EGFR and Rho/Rock signaling pathways in different cell types^74^. Importantly, all these proteins have been implicated in the development of endometriosis.

SP can also directly induce M2 polarization of inflammatory macrophages, which participate in tissue repair^75^ and are involved in promoting lesion growth and fibrogenesis in endometriosis^76,77^. Functioning through NK1R, which is widely expressed in immune cells, SP has been reported to be a potent neuroimmunomodulator^78^. SP has been shown to inhibit NK cell cytotoxicity through NK1R^79^.

SP plays an important role in cardiac fibrosis in response to hypertension^80^. Co-culture with rat primary sensory neurons from DRG and as well as SP, CGRP and vasoactive intestinal peptide or VIP facilitate fibroblasts and keratinocytes proliferation and induce collagen I production^81^. Direct contact of fibroblasts with neuronal processes promotes differentiation to myofibroblasts and their contractility^82^.

In view of the above, the results presented in this and the companion manuscript are consistent with the roles of SP/CGRP and their receptors in wound healing and fibrogenesis, and underscores the notion that sensory nerves are a notable feature of the lesional microenvironment, especially in DE lesions, and innervation plays an important role in lesional development and fibrogenesis.

We have previously shown that platelet-derived TGF-β1 promotes EMT, FMT, SMM and fibrogenesis in endometriotic lesions^21,22,83^. We have shown recently that macrophages also promote lesional fibrogenesis through identical processes^77^. We found recently that regulatory T cells also facilitate lesional fibrogenesis through the same processes (Xiao *et al*., unpublished data). In this and our *in vivo* studies^35^, we have shown that sensory nerve-derived neuropeptides also accelerate lesional fibrogenesis through similar processes. While in each case the identity of the culprit in promoting lesional fibrogenesis is different, all of them nonetheless can induce EMT, FMT, and SMM, resulting in ultimately lesional fibrosis. Hence in lesional microenvironment, there are several accessories to the crime of promoting endometriosis, who may conspire together (e.g., lesion- and/or platelet-derived TXA2 induces neurite outgrowth, and thus lesional innervation^84^) or may act independently, that together promote lesional fibrogenesis. Thus, progressive EMT, FMT, SMM and fibrogenesis jointly constitute the natural history of endometriotic lesions^27^.

DE is quite different from OE or PE^2^ and its histology resembles that of uterine adenomyosis^6^. Our findings provide an answer to a long-standing conundrum in endometriosis: Why DE tends to have more fibromuscular content than other subtypes of endometriosis? The answer simply lies in the locations of the DE lesions: unlike other subtypes, these lesions typically are in close proximity to various sensory nerve plexuses and additionally have higher density of sensory nerves than other subtypes^29,30,85,86^. Moreover, the increased density of sensory nerves in or around DE lesions also results from neurotrophic factors secreted by endometriotic lesions^84,87–90^ or platelets^84^. However, the sensory nerves in or around endometriotic lesions also become accessories to the crime in inflicting pains in women with endometriosis through accelerated lesional fibrogenesis and enhanced transduction of pain mediators to the CNS. Just as the location is vitally important to a real estate, the location of an endometriotic lesion, which defines its lesional microenvironment, is also crucially important to determine its phenotype and, consequently, symptomology. As a corollary, OE and DE, and likely other subtypes of endometriosis and adenomyosis as well^91,92^, have the identical pathophysiology and similar natural history as wounds undergoing ReTIAR. What makes them different is their locations which encompass their lesional microenvironment.

That said, some caveats should be noted. In this study, we used an endometriotic epithelial cell line instead of primary endometriotic epithelial cells due mainly to the technical difficulty in culturing the latter. In addition, we used endometrial stromal cell line (i.e. ESCs) and primary endometriotic stromal cells derived from OE (i.e. HESCs). None of these cells came from DE lesions. As such, these cells are likely to be phenotypically and functionally different from those derived from OE. However, deriving epithelial cells from DE lesions may be out of the question because of the technical difficulty and also due to the phenomenon of “stromal endometriosis”, i.e. endometriotic lesions of absent glandular epithelium^93,94^ in 12–15% of DE patients^95,96^. In addition, as DE lesions become highly fibromuscular^17^, genuine endometriotic stromal cells may be hard to found. Even one could harvest them and the resultant cells have the look and feel of endometriotic stromal cells, there is still a question as how we can be certain that they have not undergone FMT and are genuinely mesenchymal cells. Equally likely, they could have been recruited to the lesion site from elsewhere. On balance, given somewhat consistent changes in endometrial stromal (i.e. ESCs) and endometriotic stromal cells (i.e. HESCs) (see, for example, Fig. 3), our choice may be justifiable, suggesting that the phenotypic differences between OE and DE lesions may result from their respective microenvironments. This, in fact, is what this study is trying to show: sensory nerve-derived neuropeptides, which are abundant in the DE microenvironment, can turn non-DE endometriotic or even endometrial stromal cells into DE-like cells, resulting in a fibromuscular phenotype that is characteristically DE.

Our finding that sensory nerve-secreted neuropeptides promote lesional fibrosis has significant implications. First, by administration of SP and/or CGRP to mice with induced endometriosis, a mouse DE model can be developed^97^. Currently, the only animal DE model that has been reported is through grafting uterine specimens, especially in full uterine thickness, to the peritoneal cavity in baboons^98^. Issues of cost, facility and surgical skill aside, the DE mouse model can be established in as short as 3 weeks, as compared with 20–24 weeks minimum for baboon models. Obviously, the mouse DE model has many advantages over the baboon model. Moreover, if one can establish the disease model at will, the chance that we can develop novel therapeutics can be greatly increased.

Second, our results strongly suggest that NK1R may be a promising drug target for treating endometriosis through stalling lesional fibrogenesis via suppression of EMT, FMT, and SMM. Aside from delaying fibrogenesis, NK1R antagonism can also mitigate alterations in the brain induced by chronic psychological stress^99–101^, which is associated with visceral hypersensitivity and spinal NK1R up-regulation in female rodents^102,103^ and possibly in mouse with induced endometriosis as well^104–106^. Moreover, NK1R antagonism has been shown to reduce anxiety and emotional arousal circuit response to noxious visceral distension in women with irritable bowel syndrome^107^, which is likely attributable, at least in part, to increased colonic hypermotility induced by SP and NK1R activation^72,73^. Given that endometriosis can induce anxiety and depression in mouse^108^ and possibly in humans as well^109–114^, the use of NK1R inhibitors as a therapeutics for endometriosis may have added benefits.

Lastly, our study further highlights the importance of the lesional microenvironment in shaping the lesional development route and fate. Besides endometriotic epithelial and stromal cells, which traditionally have been the major research focus, other cells, such as immune cells (including platelets) and now the sensory nerve cells, are also critical aiders and abettors to the crime of inflicting pains and misery to women with endometriosis. Focusing exclusively on endometriotic cells would be difficult to explain as why OE and DE lesions differ dramatically in phenotype and symptomology and insufficient to fully understand the pathophysiology of endometriosis. In addition, the identical processes, instituted by platelets, immune cells and sensory nerves, of EMT, FMT, SMM, and fibrogenesis experienced by endometriotic cells underscore the dynamic nature in cellular identity, phenotype, function and behavior of endometriotic cells.

To conclude, we have shown that sensory nerve-derived neuropeptides SP and CGRP promote the development and fibrogenesis of endometriotic lesions through EMT, FMT and transdifferentiation to SMC. Antagonism of their respective receptors, however, stalls these processes. Consequently, sensory nerve fibers facilitate the development and fibrogenesis of endometriotic lesions along with other cells in the lesional microenvironment. Our study highlights the importance of lesional microenvironment in lesional development and fibrogenesis and also the dynamic nature of endometriotic cells. Finally, our study suggests that NK1R may be a promising therapeutic target for treating endometriosis.

## Materials and Methods

### Human samples

This study strictly adhered to the ethical principles outlined by the Helsinki Declaration and was approved by the institutional ethics review board of Shanghai OB/GYN Hospital, Fudan University. After informed consent, endometriotic tissue samples harvested for isolation and primary culture were obtained from 8 patients (mean age = 32.9 ± 5.3 years) with laparoscopically and histologically diagnosed OE but no other gynecological diseases, who had a regular menstrual cycle (proliferative phase, n = 2 and secretory phase, n = 6).

For immunohistochemistry analysis, all human tissues samples were obtained from premenopausal patients with laparoscopically and histologically diagnosed OE (n = 30) or DE (n = 30), admitted to the Shanghai OB/GYN Hospital, Fudan University, from February, 2014 to Jun, 2016. Written informed consent was obtained from all study subjects prior to sample collection. In all cases, the OEs, staged as III-IV by the revised American Society of Reproductive Medicine classification system (rASRM), were removed by stripping the cyst wall from the ovaries, and the DE samples were taken from uterosacral ligaments. For controls, endometrial tissue samples were harvested from 24 cycling women, matching, in frequency, in age- and menstrual phase with patients with OE or DE after written informed consent. These women underwent surgery because of teratoma, cervical intraperitoneal neoplasia III, and tubal ligation, but were found to be free of endometriosis, adenomyosis, and uterine abnormalities, such as uterine leiomyomas. Table 1 lists the characteristics of all recruited subjects.

**Table 1:** Characteristics of recruited patients who donated their tissue samples for this study.

Variable	Normal endometrium (n = 24)	Ovarian endometrioma (n = 30)	Deep endometriosis (n = 30)	p-value
Age (in years)				
*Mean* ± *S.D*	35.5 ± 6.4	32.8 ± 7.5	38.5 ± 6.1	0.004
*Median (Range)*	34 (26–48)	31 (23–53)	39 (27–52)	
Menstrual phase				
*Proliferative*	18 (75.0%)	13 (43.3%)	11 (36.7%)	0.084
*Secretory*	6 (25.0%)	17 (56.7%)	19 (63.3%)	
Parity				
*0*	3 (12.5%)	17 (56.7%)	7 (23.3%)	0.002
*1*	18 (75.0%)	11 (36.7%)	22 (73.3%)	
*2*	3 (12.5%)	2 (6.7%)	1 (3.3%)	
rASRM stage				
III	NA	14 (46.7%)	NA	NA
IV		16 (53.3%)		
Severity of dysmenorrhea				
*None*	24 (100.0%)	14 (46.7%)	0 (0.0%)	1.3 × 10^–14^
*Mild*	0 (0.0%)	9 (30.0%)	10 (33.3%)	
*Moderate*	0 (0.0%)	4 (13.3%)	5 (16.7%)	
*Severe*	0 (0.0%)	3 (10.0%)	15 (50.0%)	
Co-occurrence with adenomyosis				
*No*	24 (100.0%)	29 (96.7%)	16 (53.3%)	1.4 × 10^−6^
*Yes*	0 (0.0%)	1 (3.3%)	14 (46.7%)	
Co-occurrence with uterine fibroids				
*No*	24 (100.0%)	25 (83.3%)	19 (63.3%)	0.0015
*Yes*	0 (0.0%)	5 (16.7%)	11 (36.7%)	

rASRM: Revised American Society for Reproductive Medicine classification system for endometriosis.

None of the patients had received hormonal or anti-platelet treatment for at least 90 days before the surgery. In addition, none of them had any malignant or other inflammatory disease.

### Cells and reagents

Established by Strazinski-Powitz *et al*.^115^, the endometriotic epithelial cell line (HEES or 11Z)^36^ was kindly provided by Professor Jung-Hye Choi of Kyung Hee University, Seoul, Republic of Korea, and cultured in RPMI 1640 medium (Gibco Laboratories, Life Technologies, Grand island, NY, USA) supplemented with 5% fetal bovine serum (FBS) (Gibco), 100 IU/mL penicillin G, 100 μg/mL streptomycin and 2.5 μg/mL Amphotericin B (Hyclone, Utah, USA). The human endometrial stromal cell line (ESC), as reported by Krikun *et al*.^116^, was kindly provided by Professor Asgi Fazleabas of Michigan State University, Michigan, U.S.A., and cultured in Dulbecco’s modified Eagle’s medium/Ham’s F-12 medium (DMEM/F-12, Hyclone) supplemented with 5% FBS, 100 IU/mL penicillin G, 100 μg/mL streptomycin and 2.5ug/mL Amphotericin B.

The primary human endometriotic stromal cells (HESCs) were derived as previously reported^117^ and used in our previous work^22^. Briefly, the ectopic endometrial tissues were washed with DMEM/F-12 medium and minced into small pieces of about 1 mm³ in size. After enzymatic digestion of minced tissues with 0.2% collagenase II (Sigma, St. Louis, MO, USA) in a shaking bed for 1.5 h at 37 °C, they were separated by filtration through a 76-μm then a 37-μm nylon mesh. The filtrated cells were centrifuged and suspended in DMEM/F-12 medium supplemented with 10% FBS, 100 IU/mL penicillin, 100 mg/mL streptomycin and 2.5 μg/mL Amphotericin B, and seeded into 25-cm^2^ cell culture flasks and incubated at 37 °C in humidified atmosphere of 5% CO_2_ in air. The purity of endometriotic stromal cells was confirmed by immunocytochemistry using an antibody against vimentin (Abcam, Cambridge, UK), a specific marker of stromal cells, and an antibody against cytokeratin 7 (CK7) (Zhongshan Jinqiao, Beijing, China), a specific marker of epithelial cells. The vimentin staining was positive and the CK7 staining was negative after the third passage. To rule out the possibility of contamination with ovarian granulosa cells, we also stained the primary cells with follicle stimulating hormone receptor (Abcam, Cambridge, UK), a specific marker for granulosa cells^118^. The staining was found to be negative, as we previously reported^119^.

Cells with different treatments were used for quantitative real-time RT-PCR, Western blot, invasion assay, scratch test, cell immunofluorescence, cell contractility and collagen assays. SP (Sigma), a neuropeptide and inflammatory mediator involved in pain transmission, was administered at the concentration of 10^−7^ M^120^. CGRP (Sigma), a long-lasting vasodilator, was used at the concentration of 10^−7^ M following the previous report^121^. For inhibitor experiments, cells were pretreated with vehicle or the potent NK1R antagonist aprepitant (Selleckchem) (10^−6^ M)^122^ or CGRP Fragment 8–37 (CGRP 8–37) (Sigma) (10^−6^ M)^123^, a selective competitive antagonist for CGRP receptors, for 1 hour at 37 °C. Aside from the references cited above, we note that SP at the concentration of 10^−7^ M is reported to increase cell viability, reduce apoptosis, stimulate proliferation, and inducs proinflammatory signaling in some pathological conditions, such as Crohn’s disease^124^ and acute intestinal inflammation^125^. At concentrations ranged from 10^−12^ M to 10^−7^ M, CGRP is reported to significantly induce epithelial cell migration and proliferation in a dose-dependent manner^126^. CGRP at the concentration of 10^−7^ M induces complete relaxation of human coronary arteries, while pre-incubation with CGRP 8–37 at the concentration of 10^−6^ M causes almost perfectly antagonistic effect^127^. Moreover, 10^−6^ M of aprepitant, or about 534.4 ng/mL, is well within or below the plasma concentration in cancer patients who took aprepitant to prevent chemotherapy-induced nausea and vomiting: After taking one 125-mg capsule on day 1 followed by an 80-mg capsule on days 2 and 3^128^, the median and interquartile range of plasma concentration of aprepitant 1 day and 3 days were reported to be 768 ng/mL (592–949) and 915 ng/mL (563–1203), respectively^129^. With these considerations, we believe that our choice of doses are well justified.

### Isolation of DRG-derived neurons

Thirty virgin female Sprague-Dawley rats (Shanghai Center for Experimental Animals, Chinese Academy of Sciences, Shanghai, China), 4–5 weeks old, 100–120 g in bodyweight, were used for this study following the guidelines of the National Research Council’s Guide for the Care and Use of Laboratory Animals^130^ and approval by the Ethics Review Board, Shanghai OB/GYN Hospital.

After sacrifice by decapitation, the lumbar DRG was dissected, which was digested with 1 mg/mL collagenase type 1 A, 0.4 mg/mL trypsin type I, and 0.1 mg/mL DNase I (all from Sigma) in DMEM/F-12 at 37 °C for 30 min, and then triturated, as reported previously^131^. Following filtration through a 100-μm nylon mesh, the dissociated DRG neurons were plated on coverslips coated with 1 mg/mL poly-D-lysine at room temperature for 2 hours, and then cultured in DMEM/F-12 containing 10% FBS. Six hours later, the culture medium was replaced with DMEM/F-12 containing 1% N2 supplement (Life Technologies, Grand Island, NY, USA), and the neurons were maintained at 37 °C in humidified atmosphere of 5% CO_2_ for further experimentation.

### Cellular proliferation by CCK-8 assay

The 11Z cells and HESCs were seeded at a density of ∼2,000 per well in a 96-well plate and cultured in standard medium, then starved by a serum-free medium for 12 hours. The cells were incubated at 37 °C in a humidified atmosphere of 5% CO_2_. For stimulation experiment, 11Z cells were treated with vehicle, SP (10^−7^ M), CGRP (10^−7^ M), or both SP (10^−7^ M) and CGRP (10^−7^ M); for antagonist experiments, HESCs were treated with medium, DRG supernatant with or without pre-treatment of aprepitant (10^−6^ M), CGRP 8–37 (10^−6^ M), or both aprepitant (10^−6^ M) and CGRP 8–37 (10^−6^ M), for 1 hour. After 48 hours, 10 μL of CCK-8 solution (Dojindo Co., Ltd. Kumamoto, Japan) was added into each well, followed by incubation for 1–4 h, and the color of the solution was closely monitored. When the difference in the color was significant between groups, absorbance was measured at 450 nm. The experiment was done in triplicate.

### RNA isolation and real-time RT-PCR

Total RNA was isolated from 11Z cells, ESCs and HESCs after different treatments for 12 days, using TRIzol (Invitrogen, Carlsbad, CA, USA). The synthesis of cDNA was carried out using the reverse transcription kit (Takara, Takara Bio, Inc., Otsu, Shiga, Japan). The mRNA abundance was quantitated by real-time PCR using SYBR Premix Ex Taq (Takara). The expression values were normalized to the geometric mean of GAPDH measurements and the quantification of mRNA abundance was performed using the method as previously described^132^. Table 2 lists the names of genes and their primers used in this study.

**Table 2:** List of primers used in the real-time RT-PCR analysis.

Gene name	Sequence	
GAPDH	forward	5′-GCACCGTCAAGGCTGAGAAC-3′
	reverse	5′-TGGTGAAGACGCCAGTGGA-3′
Vimentin	forward	5′-GAACGCCAGATGCGTGAAATG-3′
	reverse	5′-CCAGAGGGAGTGAATCCAGATTA-3′
N-cadherin	forward	5′-ATCCTACTGGACGGTTCG-3′
	reverse	5′-TTGGCTAATGGCACTTGA-3′
Fibronectin (FN)	forward	5′-CCATCGCAAACCGCTGCCAT-3′
	reverse	5′-AACACTTCTCAGCTATGGGCTT-3′
PAI-1 (Serpine1)	forward	5′-ACCGCAACGTGGTTTTCTCA-3′
	reverse	5′-TTGAATCCCATAGCTGCTTGAAT-3′
Snail	forward	5′-TCGGAAGCCTAACTACAGCGA-3′
	reverse	5′-AGATGAGCATTGGCAGCGAG-3′
Slug	forward	5′-AAGCATTTCAACGCCTCCAAA-3′
	reverse	5′-GGATCTCTGGTTGTGGTATGACA-3′
Collagen 1A1 (COL1A1)	forward	5′-AGGGCCAAGACGAAGACATC-3′
	reverse	5′-GATCACGTCATCGCACAACA-3′
α-SMA	forward	5′-GCTTTGCTGGGGACGATGCT-3′
	reverse	5′-GTCACCCACGTAGCTGTCTT-3′
CCN2 (CTGF)	forward	5′-GGTCAAGCTGCCCGGGAAAT-3′
	reverse	5′-TGGGTCTGGGCCAAACGTGT-3′
LOX	forward	5′-TGCCAGTGGATTGATATTACAGATGT-3′
	reverse	5′-AGCGAATGTCACAGCGTA CAA-3′
Desmin	forward	5′-CATCCTCAAGAAGGTGTTGGAG-3′
	reverse	5′-CAAAGAGACGTGGGACGAGT-3′
OTR	forward	5′-GTGGTGGCAGTGTTTCAGGT-3′
	reverse	5′-CGTAGAAGCGGAAGGTGATG-3′

### Western blot analysis

Cells were scraped and their total proteins were extracted in a Radio-Immunoprecipitation Assay (RIPA) buffer (Fermentas, Thermo Fisher Scientific, Pittsburgh, PA, USA). The protein concentration was evaluated using a bicinchoninic acid (BCA) protein quantitative analysis kit (P0010S, Beyotime, Shanghai, China). Briefly, protein samples were loaded on a 10% SDS-PAGE and transferred to polyvinyl difluoride (PVDF) membranes (Bio-Rad, Hercules, CA, USA). The membranes were incubated at 4 °C overnight with the primary antibodies (listed in Table 3). After the membranes were incubated with HRP-labeled secondary antibodies at room temperature for 1 hour, the band images were developed with enhanced chemiluminescence (ECL) reagents (Pierce, Thermo Scientific, Rockford, IL, USA) and digitized on Image Quant LAS 4000 mini (GE Healthcare). Image quantification was performed with Quantity One software (Bio-Rad).

**Table 3:** List of antibodies used in the Western blot analyses, immunofluorescence and immunohistochemistry.

Antibody name	Catalog number	Vendor name and location	Concentration Immunofluorescence /Western blot
GAPDH (loading control)	5174	CST, Boston, MA, USA	−/1:1000
E-cadherin	3195 S	CST	1:100/1:1000
Vimentin	ab8978	Abcam, Cambridge, UK	1:100
Alpha smooth muscle actin (α-SMA)	ab5694	Abcam	1:100
F-actin	ab205	Abcam	1:100
Oxytocin receptor (OTR)	ab115664	Abcam	1:200
Desmin	ab6322	Abcam	1:200
Smooth muscle, myosin heavy chain (SM-MHC)	ab81031	Abcam	1:200
Neurokinin 1 receptor (NK1R)	ab183713	Abcam	1:50
Calcitonin receptor like receptor (CRLR)	ab84467	Abcam	1:200
Receptor activity modifying protein 1 (RAMP-1)	ab203282	Abcam	1:100
Calcitonin gene related peptide (CGRP)	ab47027	Abcam	1:200

### Scratch assay

Scratch assay was used to assess the migratory propensity of 11Z cells and HESCs as described previously^22^. Briefly, the cells were plated in six-well tissue culture dishes at a concentration of 1 × 10^5^ cells. After the cells reached 80–90% confluence, the tip of a micropipette was used to wound the cells, creating a linear, cross-stripe scrape ~2 mm wide. The cells were washed with PBS to remove floating cellular debris and re-fed with either serum-free medium (for use as a negative control) or experimental medium (RPMI 1640 or DMEM/F-12 containing vehicle with a combination of SP, CGRP, or both). Cell migration was photographed (Olympus BX53, Olympus, Tokyo, Japan) at 200× magnification and the corresponding distance was evaluated at 0, 12, 24, 48 hours after the scratch, and recorded with an attached digital camera (Olympus DP73, Olympus). At each time point, 2–3 measurements were carried out for each well and the average distance of each edge of cells traversed relative to the initial scratch distance was calculated in pixel values using Image Pro-Plus software 6.0 (version 6.0.0.206; Media Cybernetics, Inc, Bethesda, MD, USA). Each assay was done in triplicate. All experiments were conducted in the presence of 5 μg/mL of mitomycin-C (Sigma) to suppress cellular proliferation^133^.

### Invasion assay

To evaluate the effect of SP and/or CGRP or DRG supernatant treatment and of the SP/CGRP antagonism on the invasiveness of 11Z cells, Invasion assay with Biocoat 24-well Matrigel (BD Biosciences, Franklin Lakes, NJ, USA) invasion chambers (Corning, Tewksbury, MA, USA) was used. Briefly, ~10^5^ 11Z cells resuspended in 200 μL serum-free culture medium containing different treatments were added into each upper chamber. For the stimulation experiment, we added the vehicle, SP (10^−7^ M), CGRP (10^−7^ M), or both SP (10^−7^ M) and CGRP (10^−7^ M); for the antagonism experiment, we added the medium, DRG supernatant with or without pre-treatment with aprepitant (10^−6^ M), CGRP 8–37 (10^−6^ M), or both aprepitant (10^−6^ M) and CGRP 8–37 (10^−6^ M) for 1 hour. The lower chamber was also added with 600 μL culture medium with 20% fetal bovine serum (FBS). After incubation at 37 °C for 48 hours in a humidified atmosphere of 5% CO_2_ in air, the number of cells adhering to the lower surface of the membrane was counted under the microscope. Infiltrated cells were fixed by 95% alcohol, nuclear stained, and counted under the microscope (Olympus BX53) fitted with a digital camera (Olympus DP73). The invasion index was defined to be the average count of the infiltrated cells under 200× magnification of randomly selected 3–5 fields. All experiments were carried out in triplicates.

### Cell immunofluorescence

Endometriotic epithelial 11Z cells were seeded into 12-well plates overnight and treated with vehicle, SP (10^−7^ M) and/or CGRP (10^−7^ M) for 0, 6 or 12 days. HESCs were seeded into 12-well plates and treated with either vehicle, SP (10^−7^ M), CGRP (10^−7^ M) or DRG supernatant for 0, 4, 8, or 12 days. The cells were then washed with PBS twice, fixed with 95% ethylalcohol for 30 minutes, suspended in 0.3% Triton X-100 for 20 minutes, and blocked in 10% normal goat serum followed by incubation with the primary antibodies. For 11Z cells, E-cadherin, vimentin, F-actin and α-SMA were used to evaluate the occurrence of EMT and FMT, respectively; HESCs were incubated at 4 °C overnight with antibodies against α-SMA, F-actin, OTR, desmin, or SM-MHC. The information on these antibodies is listed in Table 3. After washing, cells were incubated at 37 °C for 1 hour with Alexa Flour 488-conjugated goat anti-mouse IgG (Abcam) or Alexa Flour (R) 647 goat anti-rabbit (Abcam) and then washed with PBS and stained with DAPI. Images of stained cells were obtained by a laser scanning confocal microscope (Leica TCS SP5 Confocal Microscope, Leica, Solms, Germany) at room temperature. Images were recorded separately with different objective lenses (20x, 40x/1.25-oil and 100x 1.4-oil objective), then exported as a TIFF-format digital file. All experiments were carried out in duplicate.

### Collagen gel contraction assay

The contractility of cells treated with different conditions was evaluated by the cellular collagen gel contraction assay kit (CBA-201, Cell Biolabs, San Diego, CA, USA) according to the vendor’s instructions. Briefly, HESCs were embedded in the collagen gel and cultured three-dimensionally. They were suspended in the collagen solution (2–5 × 10^6^ cells/mL), and the collagen/cell mixture (0.5 mL/plate) was dispensed into 24-well plates (Corning) and incubated at 37 °C for 1 hour. Immediately after collagen polymerization, 1 mL of culture medium with designated treatment was added to the top of each collagen gel lattice. After incubation for 72 hours, the collagen gels were gently released from the side of the culture dishes with a sterile spatula, and the gels were photographed and the then the diameter of each gel surface was carefully measured after release with a vernier caliper at 3, 24 and 48 hours, respectively. In case of oval-shaped gel surface, the longest and shortest diameters were measured and then averaged. The difference between the diameter of the well and the diameter of the gel surface indicates the extent of cellular contractility.

### Immunohistochemistry

Tissue samples were fixed (10% formalin (w/v)) and then paraffin embedded. Each tissue block was serially sectioned (4-μm), and the first resultant slide was stained with hematoxylin and eosin to validate pathologic diagnosis, with the subsequent slides being used for IHC analysis of α-smooth muscle actin (α-SMA) (1:100, Abcam), NK1R (1:50, Santa Cruz), CRLR (1:200, Abcam), RAMP-1 (1:100, Abcam), and CGRP (1:200, Abcam). α-SMA was stained for myofibroblasts and differentiated smooth muscle cells (SMCs), and CGRP were used as specific markers for sensory nerve fibers. The number of nerve fibers was determined as previously described^87^. The area with the greatest number of nerves was selected, and after scanning the section at low magnification (100x), five randomly selected areas were evaluated and averaged for each lesions. Negative control sections were processed by omitting the primary antibody. Dorsal root ganglia were used as positive control for the CGRP staining.

The endogenous receptor for SP is NK1R^44^, while for CGRP, its major receptors are calcitonin receptor like receptor (CRLR) and receptor activity modifying protein 1 (RAMP-1). CRLR by itself cannot function as a receptor for CGRP as it needs to form complexes with accessory proteins from the RAMP family^134^. Routine deparaffinization and rehydration were carried out, as reported previously^135^.

To retrieve antigens, the slides were heated at 98 °C in a citrate buffer (pH6.0) for 30 minutes and then cooled to room temperature. After incubation with goat blocking serum for 15 min at room temperature, the processed slides were then incubated at 4 °C overnight with the intended primary antibodies (listed in Table 3). After washing with phosphate-buffered saline, the slides were added with the horse reddish peroxidae-labeled secondary antibody Detection Reagent (Sunpoly-HII; BioSun Technology Co, Ltd, Shanghai, China) and incubated at room temperature for 30 minutes. The resultant bound antibody complexes were stained with diaminobenzidine for 3–5 minutes or until appropriate for microscopic examination, followed by counterstaining with hematoxylin (30 seconds) and then mounted. The staining images were procured with an Olympus BX53 microscope fitted with an Olympus DP73 digital camera. For each sample, 3–5 images at 400x magnification, selected at random, were taken to arrive a mean density value using the software Image Pro-Plus 6.0 (Media Cybernetics, Inc).

### Masson trichrome staining

To identify collagen fibers in endometriotic tissue samples, Masson trichrome staining was performed. The sections (4-μm, paraffin embedded) were deparaffinized in xylene and rehydrated in a series of graded alcohol, then were soaked in Bouin’s solution at 37 °C for 2 h. The Bouin’s solution was made with 75 mL of saturated picric acid, 25 mL of 10% (w/v) formalin solution and 5 mL of acetic acid. Following vendor’s instructions, the sections were processed using the Masson Trichrome Staining kit (Baso, Wuhan, China). The extent of lesional fibrosis, defined to be the areas of the collagen fiber layer (stained in blue) relative in proportion to the entire portion of the endometriotic lesions, was quantitated by the software Image Pro-Plus 6.0.

### Collagen assay

The cell culture medium was collected after 11Z cells (n = 3), ESCs (n = 3) and HESCs (n = 8) treated with different conditions for 72 hours and then subjected to Sircol soluble collagen assay (S1000, Biocolor, Carrickfergus, UK) following the manufacturer’s instructions. Briefly, the culture medium was collected and then centrifuged to discard the particulate materials on the bottom. Since the medium contained serum supplement, low protein binding microcentrifuge tubes (Eppendorf, Hamburg, Germany) were used. The absorbance value at 570 nm filter indicates the amount of collagens in the culture medium. The concentration of collagen in 1 mL culture medium was determined by the reference standard curves obtained using a microplate reader (Biotek, Winooski, VT, USA).

### Statistical analysis

To compare the distributions of continuous variables between two groups, Wilcoxon’s rank test was employed. To compare the before-after difference for the same group of subjects, the paired t-test was used for data from experiments using cell lines due to uniformity, and paired Wilcoxon test was used for data from experiments using primary cells because of possible heterogeneity. Pearson’s correlation coefficient was used for two continuous variables. When one or more variables were ordinal, Spearman’s correlation coefficient was used. The result was considered to be statistically significant when the P value was less than 0.05. All calculations were carried out using the software R (version 3.5.0)^136^.

## Supplementary information

Supplementary information
