# Supplementary material for: Neuropeptides Substance P and Calcitonin Gene Related Peptide Accelerate the Development and Fibrogenesis of Endometriosis
Source: Sci Rep. 2019 Feb 25;9:2698. doi: 10.1038/s41598-019-39170-w (PMC6389969; doi:10.1038/s41598-019-39170-w)
Supplement: Supplementary file 1 — Supplementary information [file 41598_2019_39170_MOESM1_ESM.docx]

**Supplementary Information**

**Neuropeptides Substance P and Calcitonin Gene Related Peptide Accelerate the Development and Fibrogenesis of Endometriosis**

**Dingmin Yan, M.D., Xishi Liu, M.D., Ph.D., Sun-Wei Guo, Ph.D.**

**
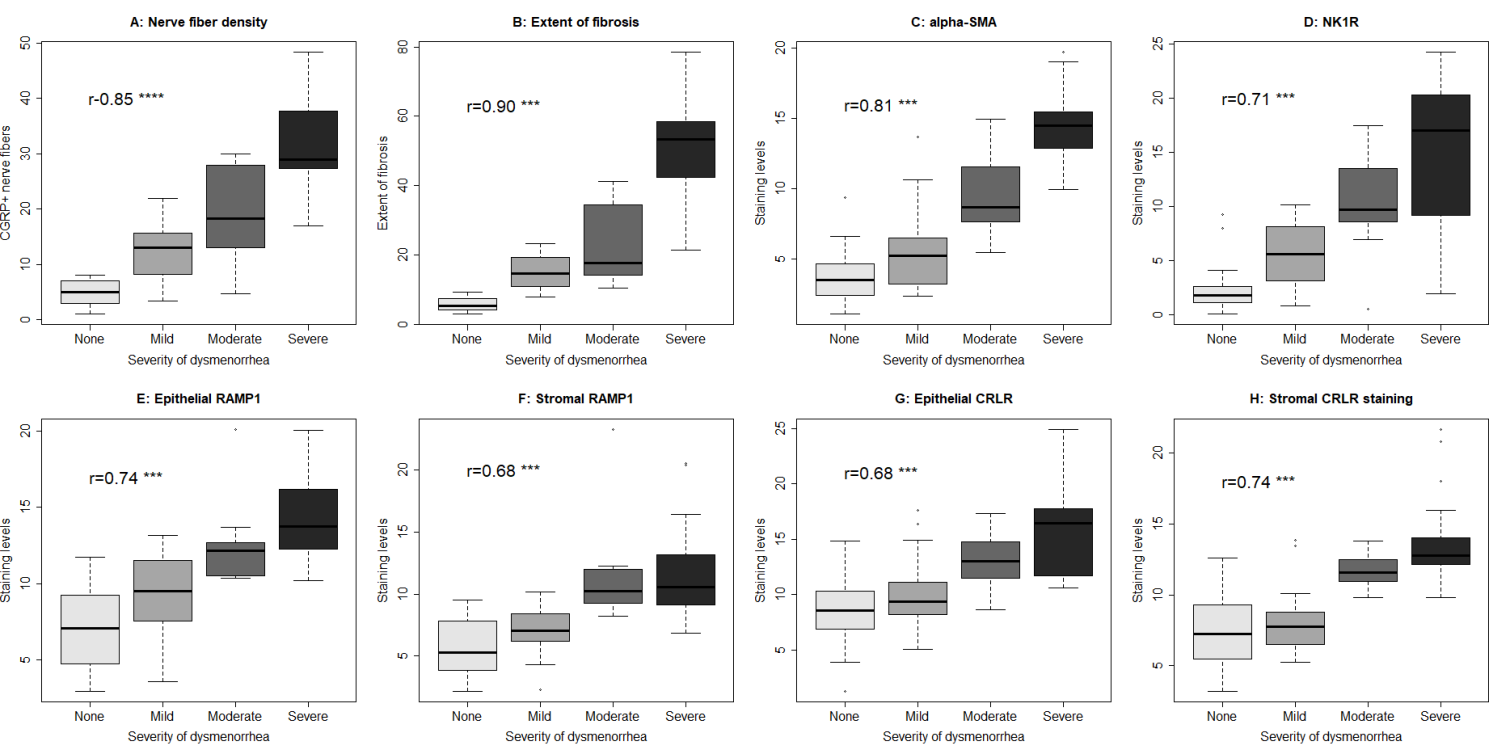
**

**Supplementary Figure S1.** Boxplots of lesional nerve fiber density (A), the extent of lesional fibrosis (B), the lesional staining levels of α-SMA (C), NK1R (D), epithelial RAMP-1 (E), stromal RAMP-1 (F), epithelial CRLR (G) and stromal CRLR in women with different severity of dysmenorrhea. The numbers shown in each figure is the Spearman’s correlation coefficient, and *** indicates that the statistical significance level of the correlation coefficient is less than 0.001.
